# Northern African strains of human T-lymphotropic virus type 1 arose from a recombination event

**DOI:** 10.1186/1742-4690-12-S1-P80

**Published:** 2015-08-28

**Authors:** A Desrames, O Cassar, O Gout, O Hermine, GP Taylor, PV Afonso, A Gessain

**Affiliations:** 1 Institut Pasteur, Unité d’Epidémiologie et Physiopathologie des Virus Oncogènes, Département de Virologie, Paris, France CNRS, UMR 3569, Paris, France; 2 Service de Neurologie, Fondation Rothschild, Paris, France; 3 Service d’Hématologie, Hôpital Necker, Paris, France; 4 Section of Infectious Diseases, Faculty of Medicine, Imperial College, London, UK

## 

Although recombination is a major source of genetic variability in retroviruses, no recombinant strain had been observed for HTLV-1, the first isolated human-pathogenic retrovirus. Different genotypes exist for HTLV-1: Genotypes b and d to g are restricted to central Africa, while genotype c is only endemic in Australo-Melanesia. In contrast, the cosmopolitan genotype A is widely distributed. We applied a combination of phylogenetics and recombination analysis approaches to a set of new HTLV-1 sequences, including the complete LTR and a 522-bp fragment of the env gene, which we collected from 19 countries throughout North, West and Central Africa, the continent where the virus has the largest endemic presence. The samples were obtained from 41 HTLV-1 infected individuals with different clinical statuses including ATL, TSP/HAM and asymptomatic carriers. The recombinant search and breakpoint detection were performed by boot scanning in Simplot to compare inferred clusters of sequences to each other. Finally, molecular clock analyses were performed to date the recombinant event observed. This led us to demonstrate the presence of recombinants in HTLV-1. Indeed, the HTLV-1 strains currently present in North Africa have originated from a recombinant event between strains from Senegal and West Africa. This recombination is estimated to have occurred around 4,0 years ago. This recombination seems to have been generated during reverse transcription. In conclusion, we demonstrate that, albeit rare, recombination can occur in HTLV-1 and may play a role in the evolution of this retrovirus (Desrames et al., J. Virology, 88, 2014). In order to precise the geographical distribution of this recombinant within the African Continent, we studied a new set of samples from HTLV-1 infected patients of diverse African origin. The data on this on-going study will be presented.

